# Climate change: why oncologists need to get involved

**DOI:** 10.1038/s44276-023-00023-9

**Published:** 2024-03-08

**Authors:** Joan H. Schiller

**Affiliations:** Oncology Advocates United for Climate and Health; former Deputy Director of Harold Simmons Cancer Center, University of Texas Southwestern(retired); University of Virginia, Lung Cancer Research Foundation, Vienna, Virginia USA

## Abstract

A warming planet will have devasting effects on human health – including the care, diagnosis, prevention, and treatment of cancer patients. As oncology health care professionals, we have a moral and professional obligation to educate our peers, health systems, the public, and other stakeholders as to the dangers they can expect, and how they can be prevented or mitigated. There are numerous ways that we, as trusted messengers, can take action, either personally, locally, nationally, or by supporting non-profit organizations advocating for climate change and cancer.

**Figure:**
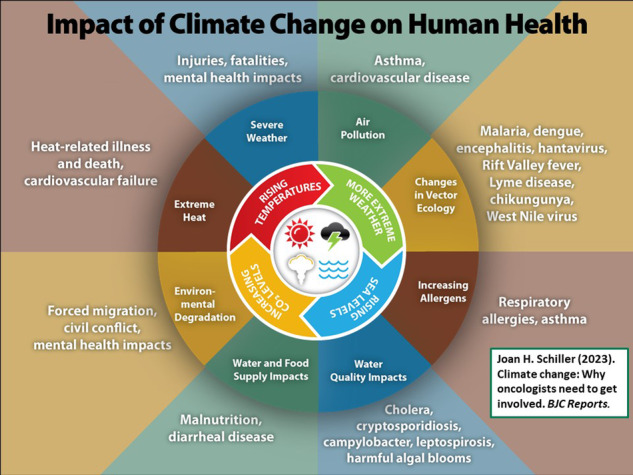
Impact of climate change on human health. Source: National Center for Environmental Health, Centers for Disease Control and Prevention, https://www.cdc.gov/climateandhealth/effects/default.htm.

Impact of climate change on human health. Source: National Center for Environmental Health, Centers for Disease Control and Prevention, https://www.cdc.gov/climateandhealth/effects/default.htm.

*“Never doubt that a small group of thoughtful committed citizens can change the world; indeed, it is the only thing that ever has.”*

Margaret Mead

Anthropologist

Awarded the Presidential Medal of Freedom, 1979

I recall a 35-year-old young woman – a never smoker, who presented with non-small cell lung cancer that had spread to her bones, brain, and other lung. She was a physician, (a gastroenterologist), mother of two, non-smoker, a long-distance runner, and died of metastatic lung cancer about three years after her diagnosis. I also remember a 32-year-old emergency room nurse who had never smoked with two teenage daughters. She was divorced, and desperately worried about what would happen to her high schoolers when she was gone. Both of these women were “lucky” in the sense that they survived long enough to see their children graduate high school and become independent adults, largely because their tumors harbored “actionable” driver mutations.

Do we know what caused lung cancer in these women, or the numerous other never-smokers I have had the privilege to care for over my career? No. What we do know is that the incidence of lung cancer in those who have never smoked is rising, particularly among young women [1] ^ii^; and that air pollution is a Class 1 carcinogen, as labelled by the International Agency for Research on Cancer (IARC) [2].

In 2021, the World Health Organization called climate change the single biggest health threat facing humanity [3]. And yet, climate change is often perceived by the public as an environmental threat or a political problem. Largely overlooked is that climate change and air pollution markedly impact cancer incidence, care delivery and outcomes.

Climate change and air pollution are two sides of the same coin; they both are due to the burning of fossil fuels. Climate change is largely attributable to the production of greenhouse gases from the burning of fossil fuels, which is also a major source of air pollutants. Issues related to burning of fossil fuels stem not just from man-made combustion. Climate change also has increased both the frequency and the intensity of forest fires and lengthened the fire season; smoke produced by these wildfires impacts air many thousands of miles away, as we have seen recently in the US when smoke from wildfires in Canada blanketed the Midwest and East Coast.

What are the impacts of climate change and air pollution on people with cancer? First of all, lets discuss the impact of climate change and health, because a warming climate will impact people, in many ways, including increases in [4]: heat related illnesses, vector-borne diseases, diarrheal diseases due to poor water quality; asthma and allergies; dehydration and renal impairment; food insecurity; and mental stress. Although all of these will impact the outcomes of people with cancer, the two most immediate impacts of climate change on cancer care are in the areas of air pollution and access to care.

## Air pollution

Ambient air pollution is blamed for 7 million deaths worldwide, one in five deaths globally [5]. The Global Burden of Disease 2017 Risk Study calls it out as the second most common cause of lung cancer, after smoking [6]. Numerous epidemiological studies have shown that people living in highly polluted areas are about 14% - 16% more likely to die of lung cancer than those who live in less polluted areas [7, 8]. Additionally, 10% - 20% cases of lung cancer worldwide occur in people who have never smoked. One meta-analysis reported that the estimated hazard ratio, adjusted for age, sex, and smoking status, was 1·13 (95% CI 1·07–1·20) per 10 μg/m³ elevation in PM2.5 [9].

Air pollution contains “fine” particulate matter 2.5 microns thick (PM2.5). Both air pollution and PM2.5 particles have been categorized as Group 1 carcinogens by the International Agency for Research and Cancer [2]. These particles penetrate deep into the lung’s terminal bronchioles, causing local inflammation, and from there translocate into the circulation, causing systemic inflammation. Although the mechanism by which PM2.5 causes lung cancer is not clear, in mice, inhalation of PM2.5 induces an inflammatory axis driven by IL1B, which is associated with the development of lung cancer. Mouse tumor formation was prevented by treatment with anti-IL1B therapy, suggesting a causal link [10].

Smoke from the burning of fossil fuels also contains gaseous pollutants (such as sulfur dioxide [SO2], nitrogen dioxide [NO2], carbon monoxide [CO], and volatile organic compounds (VOCs) such as petrochemical solvents; PAHs (e.g., benzo[a]pyrene and polar compounds), hazardous air pollutants (HAPs) including benzene and formaldehyde, among others. These molecules and compounds can generate DNA adducts, silence tumor suppressor genes, cause epigenetic modifications such as abnormal DNA methylation, chromosomal instability, transcriptional changes in microRNAs, and result in oxidative stress and an increase in free radicals [9].

## Access to care

Climate change-related extreme weather events such as hurricanes, flooding, and droughts can disable infrastructure and impair the ability to access cancer care, leading to delays and disruptions in treatment, increased morbidity, and death [11]. Disruptions include loss of power, electricity, water, radiotherapy equipment, clinical facilities, transportation, and medical records [12]. Medical shortages arise as supply chains are disrupted [11]. Many patients arrive at emergency services without their medications, oxygen, equipment, and medical records [12]. Communication breakdowns also impact workforce management and evacuation of patients and staff [12]. Research can be negatively impacted due to loss of data and biological samples, fewer studies, longer times to open studies, and longer times to accrual [11].

## Disparate impact of climate change

In the United States, vulnerable populations tend to live in communities that have a higher burden of fossil fuel infrastructure which leads to greater pollution and related health issues. Globally, low- and middle-income countries, which contribute the least to climate change, will suffer the most. Low- and middle-income countries already have lesser health infrastructures and will struggle to a greater degree than developed countries to sustain healthcare delivery under climate duress [12].

## A call to action

So why do oncology physicians, scientists, and other cancer health care professionals need to get involved? It is because cancer is a very, very scary word, and, as oncology health professionals, we are trusted messengers. A survey reported in the 2018 Lancet Countdown reported that 49% of respondents rated their primary care doctor as people they have strong or moderate trust as sources of health impacts of climate change; only 8% indicated they strongly or moderately distrusted them [13]. As health care professionals, we have a duty and a responsibility to get the word out that their health is at risk. All citizens need to know that climate change is not just an environmental hazard, or a political or social concern, but that it is a health concern, and can affect whether they develop cancer, how they are treated for it and what their outcomes will be.

What can we, as oncology health professionals, do? In addition to serving as examples by changing our personal behavior (reducing our use of combustion engines; using clean renewable power, etc.), we can educate our patients and the public by speaking out to our community (letters to our local newspapers; community events, townhall meetings, etc); to our medical colleagues (e.g. Grand Rounds, seminars) and to our hospital administrators to reduce their carbon footprint. The U.S. health care sector is estimated to contribute 8% of all US pollution [14], and 27% of the global healthcare footprint, the highest in the world [15]. We can inform our professional societies of the impact their activities and meetings have on the climate through such carbon-intensive activities as professional meetings, including airline transportation, ground transportation, food services, meeting equipment and operations, and meeting materials [16, 17]. We can tell our leaders they have a responsibility to their constituents, their constituent’s children and grandchildren, and their nation and the world by using the ballot box. We can stress that climate change is a non-partisan issue - it will affect everyone regardless of gender, race, ethnicity, socioeconomic status, geographical location, and political or religious affiliation. We can urge our pharmaceutical partners to move to carbon neutral and conduct a life cycle analysis of the carbon impact of their products. We can expose the injustice to the poor and underserved populations and countries who contribute the least to climate change yet suffer disproportionately.

Can we do this when we are we are busy and overwhelmed? In the US, the physician burnout rate spiked to 63% in 2021 [18]. Given our clinical, research, teaching, and administrative responsibilities, we are all strapped for time. Our professional work is critical, but there remain a range of ways in which we can all contribute, each taking varying amounts of time commitment. One example is to advocate through others – e.g. non-profit organizations who are already organized to carry on such work. There are many out there advocating for climate change and health care; there are relatively few, however, who advocate specially for climate activism and cancer.

**Fig. 1:**
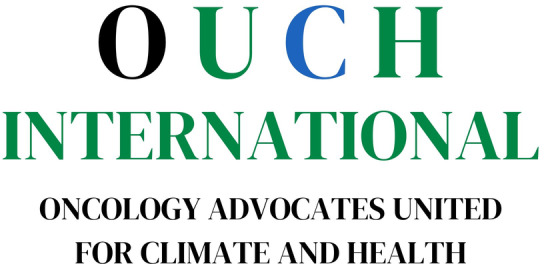
OUCH logo.

One such organization is an emerging group of oncology health professionals known as Oncologists United for Climate and Health. www.ouchforclimate.org Fig. 1. Unlike other professional cancer organizations, OUCH focuses only on the rapidly evolving health crisis. Although other professional cancer organizations also have developed policy statements or white papers regarding the impact of the climate on cancer, these organizations speak only for their members and have various other responsibilities to them. OUCH serves a unique need in which all oncology health care professionals can participate, regardless of their discipline, disease interest, specialty, locality, or whether they belong to another professional organization.

The objective of the group is to advance awareness, actions, and policies that mitigate the effects of climate change on cancer incidence, outcomes, and health equity. We educate oncology health care providers, stakeholders, and patients about the effects of climate change on their patients, practices, and communities; we engage and empower the oncology healthcare sector to expand climate action and education; and we advocate for climate change action through public policy. We do this through public testimony; by giving lectures and talks to medical and civic organizations; and working with medical and oncology related professional organizations to amplify our voices on the health and cancer consequences of climate change. We have written an open letter documenting the link between climate change and cancer, calling for a reduction in the burning of fossil fuels. It currently has been signed by cancer care providers (international physicians, scientists, nurses, pharmacists, and advocates) from 15 countries, including two professional organizations representing more than 700 members, and is attached to this paper. If you are interested in adding your name to the statement, please fill out this very brief form: OUCH’s climate & cancer sign-on.

We share the hope that collectively, we can turn the tide of climate change and the cancer-related risks that come with it.

Please join us.

Joan Schiller, MD
